# Biological Activities of *Citrus aurantium* Leaf Extract by Optimized Ultrasound-Assisted Extraction

**DOI:** 10.3390/molecules28217251

**Published:** 2023-10-24

**Authors:** Guey-Horng Wang, Chun-Ta Huang, Hsiu-Ju Huang, Chi-Hsiang Tang, Ying-Chien Chung

**Affiliations:** 1 Research Center of Natural Cosmeceuticals Engineering, Xiamen Medical College, Xiamen 361008, China; 2 Department of Biological Science and Technology, China University of Science and Technology, Taipei City 115311, Taiwanchtang2023@gmail.com (C.-H.T.)

**Keywords:** *Citrus aurantium*, resource recovery, total phenols, ultrasound-assisted extraction

## Abstract

Several studies have explored the biological activities of *Citrus aurantium* flowers, fruits, and seeds, but the bioactivity of *C. aurantium* leaves, which are treated as waste, remains unclear. Thus, this study developed a pilot-scale ultrasonic-assisted extraction process using the Box–Behnken design (BBD) for the optimized extraction of active compounds from *C. aurantium* leaves, and their antityrosinase, antioxidant, antiaging, and antimicrobial activities were evaluated. Under optimal conditions in a 150× scaleup configuration (a 30 L ultrasonic machine) of a pilot plant, the total phenolic content was 69.09 mg gallic acid equivalent/g dry weight, which was slightly lower (3.17%) than the theoretical value. The half maximal inhibitory concentration of *C. aurantium* leaf extract (CALE) for 2,2-diphenyl-1-picrylhydrazyl–scavenging, 2,2′-azino-bis(3-ethylbenzothiazoline-6-sulfonic acid)–scavenging, antityrosinase, anticollagenase, antielastase and anti-matrix metalloprotein-1 activities were 123.5, 58.5, 181.3, 196.4, 216.3, and 326.4 mg/L, respectively. Moreover, the minimal inhibitory concentrations for bacteria and fungi were 150–350 and 500 mg/L, respectively. In total, 17 active compounds were detected in CALE—with linalool, linalyl acetate, limonene, and α-terpineol having the highest concentrations. Finally, the overall transdermal absorption and permeation efficiency of CALE was 95.9%. In conclusion, our CALE demonstrated potential whitening, antioxidant, antiaging, and antimicrobial activities; it was also nontoxic and easily absorbed into the skin as well as inexpensive to produce. Therefore, it has potential applications in various industries.

## 1. Introduction

*Citrus aurantium* L. (bitter orange) is a plant belonging to the genus *Citrus* (family Rutaceae). It is native to Southeast Asia, but it has been widely planted in other regions, such as North Africa (i.e., Tunisia, Morocco, and Egypt), West Asia (i.e., Iran and Pakistan), and Western Europe (i.e., France and Italy) [1]. All parts or organs of *C. aurantium* are usable, and *C. aurantium* trees are often used for ornamental purposes. *C. aurantium* fruit are edible, whereas the derivatives of its peels, leaves, flowers, and seeds are of high value, containing various biologically active substances [2]. For instance, extracts or essential oils (EOs) from *C. aurantium* peels, flowers, and leaves are often used as spices and its seed oils are used for food preparation and plasticization [3]. *C. aurantium* flower extracts are the most valuable, used in perfumes, and applied for aromatherapy and medical purposes [3].

Appropriately processed *C. aurantium* organ extracts have antibacterial, insecticidal, antioxidant, anticancer, antianxiety, antiobesity, and antidiabetic activities [4]. Molecular, animal, and human studies have demonstrated the safety of functional ingredients in these extracts; thus, they can be used as ingredients in food and dietary supplements [5]. Studies thus far have focused on the biological activity of *C. aurantium* flowers and seeds. However, a large amount of *C. aurantium* leaves with potential active ingredients are discarded, which may pose an environmental risk [6]. *C. aurantium* leaf extract (CALE) has substantial antioxidant and antimicrobial properties [7,8]. However, CALE’s biological activity often depends on various factors, such as the origin, variety, environmental background (soil texture and degree of fertilization), and maturity stage of *C. aurantium*; the climate it is cultivated in; and the extraction methods used [8,9].

Proper extraction techniques can optimize or maximize the biological activities of CALE [10]. The 2,2-diphenyl-1-picrylhydrazyl (DPPH)- and 2,2′-azino-bis(3-ethylbenzothiazoline-6-sulfonic acid) (ABTS)-scavenging activities of the EOs extracted from the leaves of *C. aurantium* (from Tunisia) through hydrodistillation were unsatisfactory (half maximal inhibitory concentration (IC_50_) > 10,000 mg/L) [11]. Leaf EOs extracted from *C. aurantium* (from Greece) by using an identical method exhibited differences in antioxidant capacities based on the life stage of the leaves; it was 4.14 times higher in fallen leaf EOs (94.36%) than in young leaf EOs (22.79%) [12]. Tang et al. (2021) extracted the leaves of *C. aurantium* (from China) through ultrasonic-assisted extraction (UAE) with 58% ethanol as a solvent; the resulting extracts had considerably higher antioxidant activity (SOD, CAT, and GSH) than did those reported previously [8]. Moreover, *C. aurantium* leaf EOs obtained through hydrodistillation demonstrated a high minimum inhibitory concentration (MIC) for test bacteria (2700–9200 mg/L) and fungi (6900–13,000 mg/L) [11]. CALE (from Tunisia) obtained through three extraction modes in water (maceration, infusion, and decoction) demonstrated no antimicrobial activity against 12 test microorganisms [10]. For CALE (from Iran) obtained through 80% methanol extraction, MICs for *Escherichia coli*, *Staphylococcus aureus*, *Bacillus subtilis*, and *Salmonella typhi* were 150–1800 mg/L [13]. For *C. aurantium* (from India) leaf EOs obtained through hydrodistillation, MICs for *Candida albicans* were 1500–3100 mg/L [7]. Taken together, these results indicate that the biological activities of CALE depend on the *C. aurantium* origin, and the extraction method used. However, the potential physiological and pharmacological activities of CALE, particularly its bioactivity for the skin (e.g., whitening and antiaging activity), warrant further investigation.

*C. aurantium* leaves contain high-value biological compounds; thus, recycling *C. aurantium* leaf waste is a niche of product development with a potentially high return on investment, which remains under-researched. Because leaves constitute a large part of *C. aurantium* waste, extracting their active compounds using high-cost or high-tech extraction methods is unfeasible. UAE involves the use of high-frequency sound (i.e., ultrasonic) waves, which provide sufficient energy to generate strong cavitation, mechanical vibration, and disturbance effects; this increases solvent penetration and improves solvent–active compound interaction, thus accelerating the solubility of active components in the solvent [14]. According to Chemat et al. (2017), compared with conventional solvent extraction methods, UAE has the following advantages: shorter extraction time, higher active compound extraction rate, lower energy (or heat) consumption, and higher environmental friendliness (water can be used as the extraction solvent) [15].

The experimental methods used in the bioengineering process include Box–Behnken design, Plackett–Burman design, Taguchi design, full factorial design, fractional factorial design, and central composite design [16,17]. A BBD is a three-level fractional factorial design that is typically used to determine the nature of the response surface in an experimental region [18]. This design has three advantages: (i) having three levels that can be coded as −1 (low), 0 (middle), and +1 (high), (ii) creating an independent quadratic design, (iii) providing an easier way to interpret the results, and (iv) providing enough data to fit quadratic models for various applications [18,19]. For example, BBD is widely used in bioengineering processes to optimize environmental factors [20], operational factors [21], and formulation factors [22] because it can decrease the number of experimental sets.

In this study, we used UAE with water as the extraction solvent to extract active compounds from *C. aurantium* leaves. Next, we used the Box–Behnken design (BBD) combined with response surface methodology (RSM) to optimize UAE and to model the biological activity of CALE, and the optimized UAE was further verified by extracting CALE in a pilot plant.

## 2. Results and Discussion

### 2.1. Effects of Extraction Conditions on Total Phenol Content

**Figure 1:**
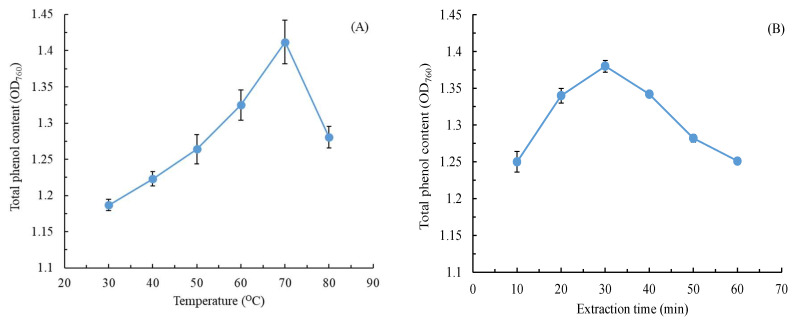

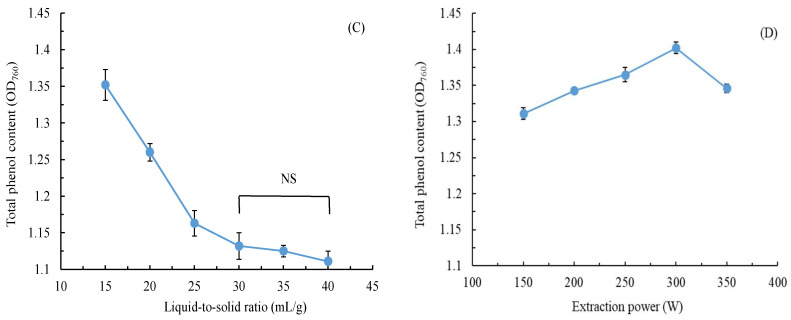
Effects of (**A**) extraction temperature, (**B**) extraction time, (**C**) liquid-to-solid ratio, and (**D**) extraction power on the TPC of *C. aurantium* leaf extract. Data are expressed as the means and standard deviations of three independent experiments. (NS = not significant *p* > 0.05).

Organic solvents (e.g., ethanol) are typically used for extracting natural products. Here, we used water as the extraction solvent to increase the environmental friendliness of our CALE extraction method. We also optimized the operating parameters such as extraction temperature, extraction time, L/S, and extraction power. The physiological activities of plant extracts often depend on the TPC [23]. Therefore, here, we used TPC as the index of extraction efficiency, which was calculated using this regression equation: *y* = 0.00925*x* + 0.0207 (*R*^2^ = 0.9981), where *x* is the gallic acid concentration (in mg/L) and *y* is OD_760_. Figure 1 illustrates the effects of various extraction conditions on the TPC of our CALE; with an increase in extraction temperature, time, and power, the TPC increased first and then decreased; the optimal extraction temperature, time, and power were 70 °C, 30 min, and 300 W, respectively. Of these three variables, extraction temperature was the most significant because, at high temperatures, phenolic compounds moved fast with notable diffusion [24]. Appropriate extraction time can improve total phenol yield; however, long extraction time will cause the decomposition of the phenolic compounds. Similar effects were observed for the ultrasonic-assisted extraction of total phenolics from *Citrus aurantium* L. blossoms [25].

A low L/S affected the mass transfer of CALE between the leaf powder and water, increasing processing costs; thus, an L/S of <15 was not considered in this study [26]. The results indicated that over an L/S range of 25–40, the TPC first decreased considerably and then gradually leveled off to a nonsignificant value (*p* > 0.05). To achieve stable operation and processing costs, an L/S of 25 mL/g was considered optimal for TPC extraction from *C. aurantium* leaves.

### 2.2. Optimization of Variables Using the BBD

Section 2.1 described a single-factor experiment for evaluating the effects of changes in one factor or parameter on the response variable (TPC). However, the effects of variables on a response variable are always interdependent and mutually restrictive [25]. The experimental factors and levels were designed on the basis of the results of the aforementioned single-factor experiment (Table 1).

Through multiple regression analysis of data from 27 experiments, we obtained a second-order polynomial equation, which revealed the following correlation of four variables with the TPC of CALE:

*y* = −19.57 + 0.4797 *x*_1_ − 0.1416 *x*_2_ + 0.0210 *x*_3_ + 0.04198 *x*_4_ − 0.003418 *x*_1_^2^ + 0.003922 *x*_2_^2^ − 0.000531 *x*_3_^2^ − 0.000077 *x*_4_^2^ − 0.001345 *x*_1_*x*_2_ + 0.000420 *x*_1_*x*_3_ + 0.000088 *x*_1_*x*_4_ + 0.000060 *x*_2_*x*_3_ − 0.000014 *x*_2_*x*_4_ − 0.000100 *x*_3_*x*_4_

Extraction temperature, L/S, and extraction power demonstrated a positive correlation with the TPC, whereas extraction time exhibited a negative correlation with the TPC. In particular, extraction temperature demonstrated the strongest correlation, whereas L/S exhibited the weakest correlation. We selected the most significant BBD-based prediction model based on the analysis of variance results (*F* = 74.07, *p* < 0.05). The coefficient value obtained (*R*^2^ = 0.9886) indicated that the model could account for 98.86% of the changes in the response variables. The fitting precision of the model met expectations, and the model fit was satisfactory. In addition, all coefficients, except for cross-product coefficients (*x*_2_*x*_3_ and *x*_2_*x*_4_), were significant (*p* < 0.05). Through RSM, we produced six sets of three-dimensional response surface plots by changing two variables; however, the remaining two variables were unchanged. The optimal extraction conditions for total phenolic content (*x*_1_ = 68.5 °C, *x*_2_ = 31.8 min, *x*_3_ = 22.1 mL/g, and *x*_4_ = 302.6 W) were obtained, and the TPC of 71.35 mg GAE/g DW was calculated from the model’s mathematical prediction. Subsequently, we performed five replicate experiments under optimal extraction conditions obtained from the flask experiment to verify the accuracy of the prediction model; a TPC of 69.09 ± 0.61 mg GAE/g DW was obtained in a pilot plant with a 150× scaleup configuration. A low deviation (3.17%) was found for the model equation, indicating that the equation was suitable for the optimization of the UAE process. The TPC of 0.062–0.792 mg GAE/g DW for CALE was obtained through maceration, infusion, and decoction [10]. The TPC of the methanolic extract of *C. aurantium* leaves has been noted to be 8 mg GAE/g DW [27]. Our results, thus, demonstrated that optimized UAE extraction considerably improves *C. aurantium* leaf waste utilization. Similar results were observed for increases in the antioxidant enzyme activity of UAE-extracted CALE [8].

### 2.3. Antioxidant Activity of CALE

**Figure 2:**
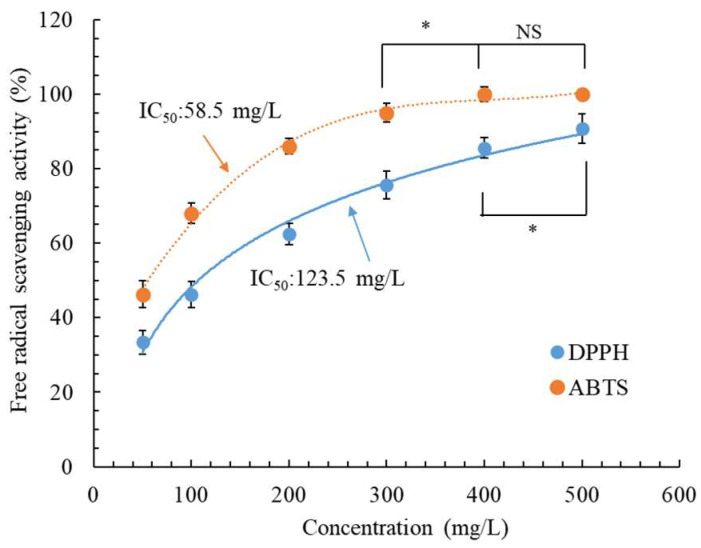
Antioxidant activity of *C. aurantium* leaf extract obtained through ultrasonic-assisted extraction under optimal conditions. Data are expressed as the means and standard deviations of three independent experiments. (NS = not significant *p* > 0.05, * *p* < 0.05).

Figure 2 illustrates two crucial antioxidant activity types of CALE. In general, the higher the free radical scavenging efficiency, the higher the antioxidant activity of the extracts. The results indicated that DPPH and ABTS scavenging activities increased as the CALE concentration increased. CALE was more effective at ABTS removal than at DPPH removal. Its IC_50_ for ABTS and DPPH scavenging was 58.5 and 123.5 mg/L, respectively. Our CALE was extracted in water; moreover, the ABTS scavenging activity assay is more suitable for measuring the antioxidant activity of hydrophilic compounds than DPPH scavenging activity [28]. Therefore, our CALE demonstrates considerable effectiveness at ABTS removal. However, leaf EOs extracted from *C. aurantium* (from Tunisia) through hydrodistillation demonstrated weak ABTS and DPPH scavenging activities (IC_50_ > 10,000 mg/L) [11], whereas those extracted from *C. aurantium* (from Morocco) through hydrodistillation demonstrated moderate DPPH scavenging activity (IC_50_: 1910 mg/L) [29]. By contrast, the ABTS and DPPH scavenging activities of CALE obtained through maceration, infusion, and decoction were 65.5–113.9 and 147.1–167.1 mg/L, respectively [10]. Therefore, our optimized UAE method demonstrated outcomes superior to those of other conventional methods.

### 2.4. Toxicity of CALE in CCD-966SK and HEMn Cells

**Figure 3:**
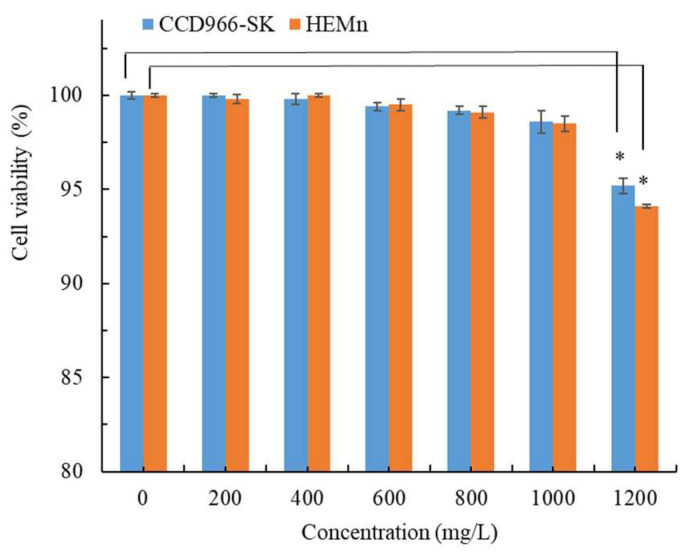
Cytotoxic effect of *C. aurantium* leaf extract obtained through ultrasonic-assisted extraction under optimal conditions in CCD-966SK and HEMn cells after 72 h treatment. Fresh medium was used as a control. Data are expressed as the means and standard deviations of three independent experiments. Significant difference was expressed by * *p* < 0.05.

In the development of new skin therapeutic substances, safety is as crucial as pharmacological efficacy. Thus, we examined the toxicity of CALE in CCD-966SK and HEMn cells by using the MTT assay. As illustrated in Figure 3, CCD-966SK and HEMn cell viability was >98.5% at a CALE concentration of ≤1000 mg/L, and nonsignificant differences (*p* > 0.05) were observed between CCD-966SK and HEMn cell viability and control cell viability. However, the cell viability differences were significant (*p* < 0.05) at a CALE concentration of 1200 mg/L. Although the CALE concentration of 1200 mg/L led to a significant reduction in cell viability compared with control cell viability, it was not toxic to the cells (94.1–95.2% viability) [30]. In a study, an inhalation test in mice demonstrated the safety of *C. aurantium* leaf EOs obtained through steam distillation [31]. These results indicate that CALE is safe for future applications in the cosmeceutical industry.

### 2.5. Analysis of Antityrosinase Activity and Melanin Content

**Figure 4:**
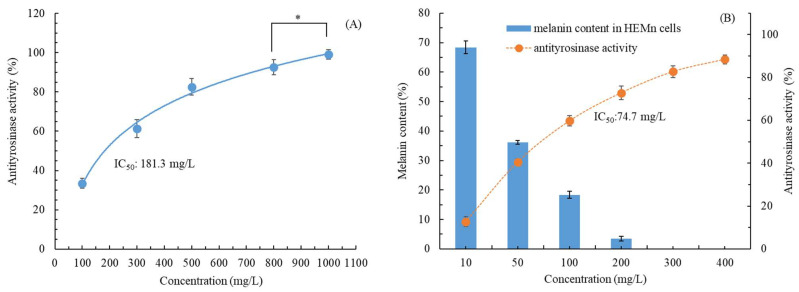
(**A**) In vitro antityrosinase activity and (**B**) antityrosinase activity and melanin content in HEMn cells after treatment with *C. aurantium* leaf extract obtained through ultrasonic-assisted extraction under optimal conditions. Data are expressed as the means and standard deviations of three independent experiments. Significant difference was expressed by * *p* < 0.05.

As illustrated in Figure 4A, the in vitro antityrosinase activity of our UAE-extracted CALE increased as the CALE concentration increased. This dose-dependent increase was also noted in cellular tyrosinase inhibitory activity (Figure 4B). Nonlinear regression analysis revealed that the cellular tyrosinase inhibitory concentration (IC_50_ = 74.7 mg/L) was higher than the in vitro tyrosinase inhibitory concentration (IC_50_ = 181.3 mg/L). El Kharraf et al. (2021) reported that *C. aurantium* leaf EOs obtained through hydrodistillation exhibited poor antityrosinase activity (34%) at 4000 mg/L [29]. As presented in Figure 4B, as the CALE concentration increased, the cellular antityrosinase activity increased gradually in HEMn cells, whereas the melanin content decreased. The antityrosinase activity of CALE was 40.5% and 72.8% at CALE concentrations of 50 mg/L and 200 mg/L (1.79 times higher), respectively, whereas the melanin content in HEMn cells was 36.1% and 3.5% at CALE concentrations of 50 mg/L and 200 mg/L (10.3 times lower), respectively. The extent of melanin production inhibition exhibited an asynchronous change, and it was much higher than that of the antityrosinase activity of CALE, suggesting that the antityrosinase mechanism of CALE is complicated, possibly involving mRNA expression, tyrosinase, related proteins, and messenger pathways [32]. Moreover, at 300 mg/L, CALE led to undetectable melanin production in HEMn cells. Taken together, these results (Figure 3 and Figure 4) demonstrate that CALE is a safe, effective whitening agent. To the best of our knowledge, this is the first study evaluating the effects of CALE on cellular antityrosinase activity and melanin production.

### 2.6. Antiwrinkle Activity of CALE

When applying plant extracts in the cosmeceutical industry, their antiwrinkle and antiaging activities in addition to the whitening effect are considered. Thus, in this study, we evaluated the anticollagenase, antielastase, and anti-MMP-1 activities of CALE. These enzymes are responsible for skin elasticity and strength and the maintenance of its flexibility [33]. Table 2 lists the IC_50_ of CALE against collagenase, elastase, and MMP-1 activities. The lower the IC_50_ value, the higher was the anticollagenase, antielastase, and anti-MMP-1 activities (196.4 ± 5.2, 216.3 ± 7.6, and 326.4 ± 9.1 mg/L, respectively). *C. aurantium* leaf EOs obtained through hydrodistillation demonstrated poor antiaging activity (IC_50_ against collagenase and elastase = 1540 and 276 mg/L, respectively) [34]. These low IC_50_ values indicate the potential application of UAE-extracted CALE in antiwrinkle skincare. Skincare products containing CALE may aid in maintaining the tenderness, smoothness, and elasticity of the skin.

### 2.7. Antimicrobial Activity of CALE

The results in the previous sections confirmed the antioxidant, whitening, and antiaging activities of CALE. However, currently, ideal cosmeceuticals include a single ingredient with multiple functions. Therefore, next, we investigated the activity of our CALE against three bacterial and two fungal strains used in USP 51 antimicrobial effectiveness tests. As listed in Table 3, the MICs for the bacterial strains ranged from 150 to 350 mg/L; CALE was most potent against the opportunistic pathogen *P. aeruginosa*. Moreover, the MFCs for the fungal strains were low (≤500 mg/L). *C. aurantium* leaf EOs have been noted to have high MICs and MFCs for *S. aureus* (8000 mg/L), *E. coli* (9040 mg/L), *P. aeruginosa* (9020 mg/L), and *C. albicans* (10,520 mg/L) [11]. CALE obtained through maceration, infusion, and decoction demonstrated no antimicrobial activity [10]. *C. aurantium* leaf EOs demonstrated antimicrobial activity, with MICs of 1300 L and 1500 mg/L against *Xanthomonas citri* (responsible for causing diseases in citrus) and *C. albicans*, respectively [7,35]. EOs extracted from the leaves of *C. aurantium* (from a distinct ecoregion: Mascarene Islands) obtained through hydrodistillation demonstrated an MFC of 4000 mg/L for *C. albicans* [34]. These results indicated that our UAE-extracted CALE has good inhibitory activity against the tested bacteria and fungi, and the differences in antimicrobial activity may be attributable to the nature of the phytoconstituents in the extracts. Thus, CALE—a single substance with multifunctional effects—can be applied as an antiseptic ingredient in various industries.

### 2.8. Analysis of Active Compounds of CALE

The area of cultivation and extraction methods are key factors affecting the chemical compositions and biological activities of CALEs [36]. Table 4 lists the composition of the main chemicals and their relative contents in UAE-extracted CALE under optimal conditions. A mere percentage of compounds >0.5% in the analyzed CALE was listed in Table 4, in which the total percentage of compounds accounted for 99.2% of the analyzed CALE. In total, 17 compounds were identified, with linalool (30.46%), linalyl acetate (13.18%), limonene (9.28%), and α-terpineol (9.25%) as the major constituents. Oxygenated monoterpenes (linalool, α-terpineol, nerol, linalyl acetate, neryl acetate, and geranyl acetate), monoterpene hydrocarbons (α-pinene, sabinene, β-pinene, β-myrcene, limonene, *cis*-β-ocimene, trans-β-ocimene, and δ-3 carene), and sesquiterpenes (*trans*-caryophyllene, α-bisabolene, and nerolidol) accounted for 63.46%, 28.42%, and 8.12% of the compounds, respectively. CALE contained the most oxygenated monoterpenes, followed by monoterpene hydrocarbons and sesquiterpenes. Sarrou et al. (2013) obtained a similar result [12]. Moreover, linalool (36.03%), linalyl acetate (23%), and α-terpineol (12.89%) were the most abundant in old leaves of *C. aurantium* (from Greece) extracted through hydrodistillation [12]. CALE (from Iran) obtained through hydrodistillation also consisted of 11 of the same compounds identified in this study; however, the most abundant compound was linalyl acetate (43.7%) and not linalool [35]. The same compounds, except α-bisabolene and nerolidol, were also found in CALE (from Italy) obtained through steam distillation; however, the most abundant compound was linalyl acetate (73.1%) in the AACNR26 clone [37]. Ellouze et al. (2012) described that linalool (43.2% to 65.97%), linalyl acetate (0.77% to 24.77%), and α-terpineol (9.29% to 12.12%) were the main components in EOs extracted through hydrodistillation from the leaves of *C. aurantium* (from Tunisia) at different harvest time points [11]. We detected large amounts of limonene and the presence of α-bisabolene and nerolidol in our CALE; this finding is in contrast with the results of studies using conventional hydrodistillation as the extraction method; however, the constituents were similar to those obtained through microwave distillation [38]. This outcome may be due to the cultivation area, the environment, the extraction method used, and the samples (fallen leaves) being different between our study and the previous study. Of all CALE constituents, α-pinene limonene, linalool, α-terpineol, nerol, and linalyl acetate demonstrated satisfactory antioxidant activity [12,29,39,40]. Linalool has potential antibacterial, antityrosinase, and antiaging activities [35,41,42]; moreover, α-pinene *cis*-β-ocimene, limonene, α-terpineol, and linalyl acetate have antibacterial and anticandidal effects [43,44]. α-Pinene, limonene, and α-terpineol exhibit significantly reduced tyrosinase activity [45,46]. Moreover, α-pinene and limonene exhibit anticollagenase and antielastase activities [47]. Because of the presence of these main components, CALE demonstrates multiple bioactivities (e.g., antioxidant, whitening, antimicrobial, and antiaging activities).

### 2.9. Transdermal Absorption and Permeation of CALE

Human skin is a dynamic and complex organ with a unique structure. From outside to inside, its basic structure can be divided into the epidermis, dermis, and subcutaneous tissue [48]. Melanocytes are mainly located in the stratum basale of the epidermis; moreover, fibroblasts—responsible for mucopolysaccharide, collagen, and elastin synthesis and secretion—are located in the dermis [49]. Only cosmeceutical ingredients that demonstrate percutaneous penetration and reach the stratum basale and dermis can exert whitening and antiaging activities. However, this phenomenon is often neglected in most cosmeceutical research; therefore, the already available cosmeceutical ingredients do not demonstrate efficient outcomes. The Strat-M membrane (a porous layer resembling the epidermis and dermis layers) demonstrates penetration behavior extremely similar to that of human skin; therefore, it can be used as a substitute membrane for human skin in experiments [50]. As listed in Table 5, 2.7% and 11.4% of our CALE were blocked and retained by the artificially simulated membrane, respectively, whereas 84.5% penetrated across the membrane. The total transdermal absorption and permeation efficiency was 95.9%. The permeation efficiency of CALE was superior to that of gels (73.8%) and emulsions (83.9%) containing 1% apple extract after a 6 h permeation period [51]. The high efficiency may be due to the small size of the compounds present in CALE (molecular weight <230). The result demonstrated that CALE efficiently acts on the target cells to exert potential whitening and antiaging effects. However, further preclinical and clinical studies investigating the topical delivery performance of CALE in commercial products (e.g., gels, emulsions, and emulgels) are warranted.

## 3. Materials and Methods

### 3.1. Chemicals, Microbial Strains, and Cells

We purchased mushroom tyrosinase, collagenase, elastase, MMP-1 protein, and chemicals (purity > 99%) from Sigma-Aldrich Co. (St. Louis, MO, USA); a Strat-M membrane from Merck Millipore (Burlington, MA, USA); tryptone soy broth (TSB), yeast malt broth (YM Broth), and potato dextrose broth (PDB) from DIFCO (Tucker, GA, USA) and Medium 254 and minimum essential medium (MEM) from Invitrogen (Waltham, MA, USA). Moreover, from the Bioresource Collection and Research Center (Hsinchu, Taiwan), we procured three bacterial strains (*E. coli* (ATCC 8739), *S. aureus* (ATCC 6538), and *P. aeruginosa* (ATCC 9027)), two fungal strains (*C. albicans* (ATCC 10231) and *Aspergillus brasiliensis* (ATCC 16404)), and a normal human skin fibroblast cell line (CCD-966SK). Finally, we obtained human epidermal melanocyte (HEMn) cells from Cascade Biologics (Portland, OR, USA).

### 3.2. Plant Material

We collected fallen leaves of *C. aurantium* L. cultivated in the mountainous area (altitude, 500 m) of Miaoli County, Taiwan, in October and November 2021. *C. aurantium* was identified by Professor Bau-Yuan Hu and a voucher specimen (20210208) was deposited in the herbarium of China University of Science and Technology, Taiwan. The collected leaves were washed with distilled water, air-dried in shade, pulverized into powder, and sifted through a 35-mesh screen. This dry, sifted powder was stored in a desiccator until further use. The moisture content of the air-dried sample was 54.3%. The dried powder was adopted in the extracting process. To evaluate its biological activity, our UAE-extracted CALE was frozen and lyophilized using a shelf freeze dryer (Uniss Corp., Taipei City, Taiwan). The moisture content of the lyophilized products was quite low and negligible.

### 3.3. UAE of CALE

UAE was performed in a digital ultrasonic machine (PS-100AL; capacity, 30 L; frequency, 40 kHz) (Shenzhen Kejie Ultrasonic Technology Co., Shenzhen, China). To extract active compounds from *C. aurantium* leaves, constant amounts (g) of dried powder and deionized water (200 mL) were mixed in a 500 mL volumetric flask and sonicated in 30 L ultrasonic machine. In principle, the height of the deionized water filled in the ultrasonic machine was to cover the height of the water in the flask, which was about 3 cm height. We used deionized water as the extraction solvent to ensure the environmental friendliness of the process.

We used single-factor experiments to evaluate the effects of extraction temperature, extraction time, liquid-to-solid ratio (L/S), and extraction (ultrasonic) power on phenolic compound extraction from *C. aurantium* leaves. In the experiments conducted for evaluating the effects of extraction temperature, we used extraction temperatures in the range of 30–80 °C, with the L/S, extraction time, and extraction power controlled at 20 mL/g (10 g of sample was dissolved in 200 mL of deionized water), 30 min, and 200 W, respectively. In the experiments conducted for evaluating the effects of extraction time, we used extraction time in the range of 10–60 min, with the L/S and extraction power controlled at 20 mL/g and 200 W, respectively, and the extraction temperature was set according to the results of the previous experiment. In the experiments conducted for evaluating the effects of L/S, we used L/S values in the range of 15–40 mL/g (5–13.3 g of sample were dissolved in 200 mL of deionized water), with extraction power controlled at 200 W, and the extraction temperature and time were set according to the results of the previous experiments. In the experiments conducted for evaluating the effects of extraction power, we used extraction power in the range of 150–350 W, and the extraction temperature, time, and L/S were set according to the results of the previous experiments. The effectiveness of CALE active ingredient extraction is expressed in terms of the total phenolic content (TPC).

Next, we assessed the combined effects of the operating parameters (extraction temperature (*x*_1_), extraction time (*x*_2_), L/S (*x*_3_), and extraction power (*x*_4_)) on phenolic compound extraction from *C. aurantium* leaves and optimized the extraction process by using the BBD at three levels (−1, 0, +1). In total, 27 experimental runs were conducted and were further modeled through RSM. The statistical significance of the produced models was evaluated through ANOVA. Design-Expert 10 (Stat-Ease Inc., Minneapolis, MN, USA) was used for designing the experiments and statistical analysis. To evaluate the feasibility of optimized UAE for total phenolic extraction, we used a pilot plant with a 150× scaleup configuration. In the scaleup experiment, 1.5 kg of pulverized leaves were dissolved in 30 L of deionized water and directly poured into 30 L ultrasonic machine.

### 3.4. Determination of TPC from CALE

TPC was spectrophotometrically determined using the Folin–Ciocalteu colorimetric method, as described by Kujala et al. (2000) with a slight modification [52]. In brief, 100 μL of our CALE solution or standard gallic acid (10–200 mg/L) was mixed with 2 mL of 2% Na_2_CO_3_ for 5 min; thereafter, 100 μL of 1 N Folin–Ciocalteu reagent was added. After 30 min incubation in the dark, the mixture was centrifuged at 3000× *g* for 15 min, and the absorbance of the supernatant was determined at 760 nm on an ultraviolet–visible (UV–vis) spectrophotometer (ThermoFisher Scientific, Waltham, MA, USA). Here, gallic acid was used as the standard. The TPC of CALE is expressed as optical density at 760 nm (OD_760_) or in milligram gallic acid equivalents (mg GAE) per gram dry weight (g DW). TPC of CALE was calculated as follows.

(1) $$\;TPC=\frac{C\times V}{M}$$

where C is gallic acid concentration (mg/L) obtained from calibration curve; V is volume of CALE taken (L); M is dried weight of CALE (g).

### 3.5. Analysis of Antioxidant Activity of CALE

The antioxidant activity of our CALE was determined in terms of its DPPH and ABTS scavenging activities. To determine DPPH scavenging activity, 1 mL of CALE at different concentrations was mixed with 1 mL of ethanol and 0.5 mL of 0.1 mM DPPH at 25 °C for 30 min in the dark; then, absorbance was read at 517 nm on a UV–vis spectrophotometer [53]. The DPPH scavenging activity of CALE was calculated as follows.

(2) $$\mathrm{DPPH}\;\mathrm{scavenging}\;\mathrm{activity}\;\left(\%\right)=\left[1-\left(\frac{{OD}_{517}\;\mathrm{of}\;\mathrm{sample}}{{OD}_{517}\;\mathrm{of}\;\mathrm{control}}\right)\right]\times 100$$

A sample without CALE was used as the control, and IC_50_ was determined at 50% scavenging activity of CALE.

To determine ABTS scavenging activity, we first prepared an ABTS reaction solution by mixing 7 mM ABTS with 2.45 mM K_2_S_2_O_8_ at a ratio of 1:1, and the mixture was kept in the dark for 16 h; this reaction solution was diluted with ethanol to yield an OD_734_ of 0.7 ± 0.02. Thereafter, 180 μL of this reaction solution was mixed with 20 μL of CALE at different concentrations in the dark. After 30 min incubation, the OD_734_ of these mixtures was measured on an Epoch enzyme-linked immunosorbent assay (ELISA) reader (BioTek Instruments, Santa Clara, CA, USA) [54]. The ABTS scavenging activity of CALE was calculated as follows.

(3) $$\mathrm{ABTS}\;\mathrm{scavenging}\;\mathrm{activity}\;\left(\%\right)=\left[1-\left(\frac{{OD}_{734}\;\mathrm{of}\;\mathrm{sample}}{{OD}_{734}\;\mathrm{of}\;\mathrm{control}}\right)\right]\times 100$$

### 3.6. Analysis of CALE Cytotoxicity

To evaluate the toxicity of CALE in CCD-966SK and HEMn cells, we conducted the 3-(4,5-dimethylthiazol-2-yl)-2,5-diphenyltetrazolium bromide (MTT) assay [55]. CCD-966SK and HEMn cells were cultured in MEM containing 10% FBS and in Medium 254 with human melanocyte growth supplement in a 96-well plate, respectively. After 24 h incubation, the cells (3 × 10^5^ cells/well) were treated with fresh medium (control) or CALE (200–1200 mg/L) for 72 h. Next, we removed the medium, washed the attached cells with phosphate-buffered saline (PBS), and added fresh medium and 20 μL of 0.02% MTT. This was followed by incubation at 37 °C under 5% CO_2_ for 4 h. Next, we discarded the supernatants and added 150 μL of dimethyl sulfoxide (DMSO) to the wells to dissolve the MTT crystals formed. The OD570 of the solution was measured on the Epoch ELISA reader. The cell viability was calculated as follows.

(4) $$\mathrm{Cell}\;\mathrm{viability}\left(\%\right)=\left[1-\left(\frac{{OD}_{570}\;\mathrm{of}\;\mathrm{sample}}{{OD}_{570}\;\mathrm{of}\;\mathrm{control}}\right)\right]\times 100$$

### 3.7. Analyses of Antityrosinase Activity and Cellular Melanin Content

In vitro mushroom tyrosinase activity inhibition of our CALE was evaluated using a method reported by Wang et al. (2017) [55]. In brief, the freeze-dried CALE powder was dissolved in 0.1% DMSO. Subsequently, 30 μL of this CALE solution at different concentrations were sequentially mixed with 970 μL of 0.05 mM PBS (pH 6.8), 1 mL of 100 mg/L tyrosine solution, and 1 mL of 350 unit/mL mushroom tyrosinase solution in dark. After 20 min of mixing, we measured OD_490_ on a UV–vis spectrophotometer. The antityrosinase activity of CALE was calculated as follows:

(5) $$\mathrm{Antityrosinase}\;\mathrm{activity}\;(\%)=\frac{\left[(A-B)-(C-D)\right]}{\left(A-B\right)}\times 100$$

where A is the absorbance without the CALE, B is the absorbance without the CALE and tyrosinase, C is the absorbance with the CALE and tyrosinase, and D is the absorbance without the tyrosinase.

In vivo tyrosinase activity inhibition of our CALE and the resulting melanin content in HEMn cells were evaluated using a method reported by Wu et al. (2021) [56], with slight modifications. In brief, HEMn cells were incubated at 37 °C under 5% CO_2_. After 24 h incubation, the cells (3 × 10^5^ cells/well) were treated with CALE at different concentrations in a 96-well culture plate for 24 h. Subsequently, the cells were washed with PBS, lysed using a cell lysis solution (1% Triton X-100, 0.1 M PBS, and protease inhibitors), sonicated for 5 min, and finally recovered through centrifugation at 8000× *g* for 15 min. To analyze tyrosinase activity in HEMn cells, the lysates were reacted with 2.5 mM l-3,4-dihydroxyphenylalanine (L-DOPA) for 1 h. Next, the OD_475_ of the solution was measured on the Epoch ELISA reader.

To determine the melanin content in HEMn cells after CALE treatment, the cells were first reacted with trypsin and then centrifuged. After the removal of the supernatant, 1 N NaOH containing 10% DMSO was added to solubilize the cell pellet at 70 °C for 1.5 h. After the cooling down of the solution, we measured the OD_405_ of the solution on the Epoch ELISA reader. Next, we quantified the melanin content in HEMn cells by using a calibration curve of synthetic melanin OD_405_ versus synthetic melanin concentrations.

### 3.8. Analysis of Antiwrinkle Activity of CALE

To analyze the antiwrinkle or antiaging activity of CALE, we measured its collagenase, elastase, and matrix metalloprotein-1 (MMP-1) activities. To measure collagenase activity, we used the fluorogenic dye-quenched (DQ) gelatin method of Vandooren et al. (2011) [57], with modifications. In brief, CALE at different concentrations was mixed with 1 unit/mL collagenase and 15 μg/mL DQ gelatin and then reacted for 20 min. Finally, we determined the solution’s gelatin proteolysis rate or collagenase activity by measuring absorbance at an excitation wavelength (OD_485_) and an emission wavelength (OD_528_) on a Synergy 2 microplate reader (BioTek Instruments, Santa Clara, CA, USA).

To measure elastase activity, CALE at different concentrations was mixed with 0.3 unit/mL porcine pancreatic elastase solution and 7 mM MeOSuc-Ala-Ala-Pro-Val-pNA and incubated at 37 °C for 15 min in a 96-well plate. The OD_405_ of the mixture was measured on the Epoch ELISA reader [58].

To analyze MMP-1 activity in vivo, we cultured CCD-966SK (4 × 10^5^ cells/well) with CALE at different concentrations for 24 h in a 96-well plate. Subsequently, the solution was reacted with a human MMP-1 ELISA kit at 25 °C for 2 h (RayBiotech, Norcross, GA, USA). Finally, the OD_420_ of the mixture was measured on the Epoch ELISA reader [59].

### 3.9. Analysis of Antimicrobial Activity of CALE

MICs of CALE for *S. aureus*, *E. coli*, and *P. aeruginosa* were determined using the dilution tube method. In brief, 10 μL of inoculum (2 × 10^8^ CFU/mL), 1 mL of CALE (at different concentrations), and 1 mL of sterile TSB were mixed in a test tube and incubated aerobically at 35 °C for 24 h. Next, the MICs were determined on basis of the measurement of the OD_600_ of the bacterial suspension on a UV–vis spectrophotometer.

Minimum fungicidal concentration (MFC) of our CALE for *C. albicans* and *A. brasiliensis* was determined using the standard plate count method. In brief, 500 μL of CALE (at different concentrations) and 50 mL of broth (YM broth for *C. albicans* or PDB for *A. brasiliensis*) containing inocula (3 × 10^5^ CFU/mL of *C. albicans* or 4 × 10^5^ spores/mL of *A. brasiliensis*) were cultured in a 125 mL conical flask and incubated at 25 °C for 3 days (*C. albicans*) or 7 days (*A. brasiliensis*). After incubation, we used serial dilution and considered the lowest concentration of CALE that led to no visible growth on the Petri dish to be the MFC.

### 3.10. Analysis of Active Compounds in CALE

The CALE was obtained through UAE in a in 150x scaleup configuration 30 L ultrasonic machine under optimal extraction conditions. The bioactive constituents of CALE were characterized through gas chromatography–mass spectrometry (GC-MS) on Shimadzu’s gas chromatograph–mass spectrometer (QP 2010, Kyoto, Japan) equipped with a DB-5 fused capillary column (30 m × 0.25 mm × 0.25 μm). The lyophilized UAE-extracted CALE was dissolved in 1 mL of methanol. Chromatographic conditions: 1 μL injection volume, split ratio of 15:1, using He as carrier gas at 1.40 mL/min; injector temperature 280 °C. Oven temperature program: 50 °C initially for 3 min, ramped at a rate of 5 °C/min to 300 °C and then kept constant at 300 °C for 10 min. MS scan conditions: interface temperature 280 °C, ion source temperature 220 °C, EI mode 70 eV, mass scan range 35–500 *m*/*z*, and scan time 0.50 s [60]. Linear retention indices were calculated with reference to n-alkanes (C_6_–C_22_), obtained from Sigma-Aldrich, and GC-MS was run under the aforementioned chromatographic conditions. All the identified components were performed in triplicate and quantified using a percentage relative peak area. Individual compounds were identified by referring to the National Institute of Standards and Technology (NIST 20) GC-MS libraries and were confirmed through comparison of their retention indices with those of authentic compounds.

### 3.11. Analyses of Transdermal Absorption and Permeation of CALE

We used a Franz-type vertical diffusion cell to conduct a percutaneous absorption test. First, we added 1 mL of 500 mg/L CALE and 6 mL of deionized water to the donor and receptor chambers, respectively. A Strat-M membrane (effective diffusion area, 1.77 cm^2^), mimicking human skin, was used as the diffusion membrane. The receptor chamber temperature was controlled at 32 °C. The receiver solution was agitated using a magnetic stirrer with a stirrer bar throughout the experiments. To evaluate the effect of CALE on skin absorption and permeation, the TPCs of the solution in the donor chamber, receptor chamber, and receptor membrane were each analyzed after a 6 h permeation period [61].

## 4. Conclusions

In this study, we developed an optimized UAE process with water as the extraction solvent to effectively extract functional ingredients with several biological activities from *C. aurantium* leaves. The CALE concentration of ≤500 mg/L provided efficient antioxidant, antityrosinase, antiaging, and antimicrobial activities as well as transdermal absorption and permeation, without toxicity to skin cells. These biological activities of our CALE were attributable to the presence of linalool, limonene, and α-terpineol. Moreover, its extraction process is low-cost and eco-friendly. To the best of our knowledge, the current study is the first to reveal the multifunctional bioactivities of CALE extracted under pilot-scale operating conditions. This CALE can be safely and efficiently applied as an ingredient in health foods, drugs, and skin cosmetics.

## Figures and Tables

**Table 1:** Response surface test factor level table.

		Coded and Actual Levels		
Variable	Variable Code	–1	0	1
Extraction temperature (°C)	*x*_1_	65	70	75
Extraction time (min)	*x*_2_	25	30	35
Liquid-to-solid ratio (mL/g)	*x*_3_	20	25	30
Extraction power (W)	*x*_4_	250	300	350

**Table 2:** Antiwrinkle activity (IC_50_ values) of *C. aurantium* leaf extract obtained through ultrasonic-assisted extraction under optimal conditions.

	Collagenase Activity	Elastase Activity	MMP-1 Activity
IC_50_ (mg/L)	196.4 ± 5.2	216.3 ± 7.6	326.4 ± 9.1

**Table 3:** Minimum inhibitory concentration (MIC) and minimum fungicidal concentration (MFC) of *C. aurantium* leaf extract obtained through ultrasonic-assisted extraction under optimal conditions against various bacteria and fungi.

MIC (mg/L)			MFC (mg/L)	
*S. aureus*	*E. coli*	*P. aeruginosa*	*C. albicans*	*A. brasiliensis*
350 ± 25 a	200 ± 20 b	150 ± 10 c	500 ± 20 d	500 ± 30 d

In each row different letters (a–d): mean significant differences *p* < 0.05.

**Table 4:** The composition of the main chemicals and their relative contents of *C. aurantium* leaf extract obtained through ultrasonic-assisted extraction under optimal conditions.

No.	RI	Chemical Compounds	Chemical Formula	Relative Content (%)
1	934	α-pinene	C_10_H_16_	1.17 ± 0.02%
2	972	sabinene	C_10_H_16_	2.31 ± 0.03%
3	978	β-pinene	C_10_H_16_	3.26 ± 0.09%
4	992	β-myrcene	C_10_H_16_	3.06 ± 0.05%
5	1028	limonene	C_10_H_16_	9.28 ± 0.26%
6	1042	*cis*-β-ocimene	C_10_H_16_	2.04 ± 0.02%
7	1050	*trans*-β-ocimene	C_10_H_16_	4.17 ± 0.08%
8	1056	δ-3 carene	C_10_H_16_	3.13 ± 0.06%
9	1100	linalool	C_10_H_18_O	30.46 ± 1.05%
10	1188	α-terpineol	C_10_H_18_O	9.25 ± 0.12%
11	1226	nerol	C_10_H_18_O	2.17 ± 0.08%
12	1257	linalyl acetate	C_12_H_20_O_2_	13.18 ± 0.68%
13	1365	neryl acetate	C_12_H_20_O_2_	3.08 ± 0.05%
14	1386	geranyl acetate	C_12_H_20_O_2_	5.32 ± 0.10%
15	1417	*trans*-caryophyllene	C_15_H_24_	3.19 ± 0.03%
16	1468	α-bisabolene	C_15_H_24_	3.28 ± 0.06%
17	1562	nerolidol	C_15_H_26_O	1.65 ± 0.01%

**Table 5:** The in vitro penetration of *C. aurantium* leaf extract across Strat-M membrane by using static Franz-type diffusion cells.

Amount Recovery (% of Applied Dose)			
Donor	Strat-M^®^ Membrane	Receptor	Total
2.7	11.4	84.5	98.6

## Data Availability

Not applicable.
